# Investigating problem-posing during math walks in informal learning spaces

**DOI:** 10.3389/fpsyg.2023.1106676

**Published:** 2023-03-06

**Authors:** Min Wang, Candace Walkington

**Affiliations:** Department of Teaching and Learning, Southern Methodist University, Dallas, TX, United States

**Keywords:** problem-posing, mathematics education, online learning, informal learning, math walk

## Abstract

Informal mathematics learning has been far less studied than informal science learning – but youth can experience and learn about mathematics in their homes and communities. “Math walks” where students learn about how mathematics appears in the world around them, and have the opportunity to create their own math walk stops in their communities, can be a particularly powerful approach to informal mathematics learning. This study implemented an explanatory sequential mixed-method research design to investigate the impact of problem-posing activities in the math walks program on high school students' mathematical outcomes. The program was implemented during the pandemic and was modified to an online program where students met with instructors *via* online meetings. The researchers analyzed students' problem-posing work, surveyed students' interest in mathematics before and after the program, and compared the complexity of self-generated problems in pre- and post-assessments and different learning activities in the program. The results of the study suggest that students posed more complex problems in free problem-posing activities than in semi-structured problem-posing. Students also posed more complex problems in the post-survey than in the pre-survey. Students' mathematical dispositions did not significantly change from the pre-survey to post-survey, but the qualitative analysis showed that they began thinking more deeply, asking questions, and connecting school content to real-world scenarios. This study provides evidence that the math walks program is an effective approach to informal mathematics learning. The program was successful in helping students develop problem-posing skills and connect mathematical concepts to the world around them. Overall, “math walks” provide a powerful opportunity for informal mathematics learning.

## 1. Introduction

Much of the research in informal math learning has examined how people use math in their everyday lives and careers (e.g., Nunes et al., 1993; Civil, 2007; Walkington et al., 2014). There is a lack of research on mathematics in designed informal learning environments (Pattison et al., 2017), although this is a growing area of interest (Mokros, 2007). Such environments include museum exhibitions, libraries, and online games. Research suggests that although visitors are often unaware that they are engaging with math when in informal settings, promising mathematical thinking and social interactions can emerge (Pattison et al., 2017). Learning in informal environments often involves developing positive attitudes, enculturation, and socialization.

This is contrasted with formal settings, where learners may see mathematics as disconnected from their lives and daily activities (Mitchell, 1993; McCoy, 2005) and wonder, “When am I ever going to use this?” (Chazan, 1999). As mathematics becomes more complex and abstract, teachers in formal settings struggle to facilitate learning experiences that address this question (Gainsburg, 2008; Walkington and Bernacki, 2014). Accordingly, research has documented the incredible difficulty that learners have to make connections between math and the real world (e.g., Saxe, 1988; Lave and Wenger, 1991; Masingila et al., 1996; Inoue, 2005). Because of this, mathematics educators face a challenging question: How can we engage learners and allow them to see that mathematics is a rich and dynamic subject they can use to describe and understand their world? Leveraging mathematical reasoning as it happens in *informal* spaces can be a way to help students make these connections, and thus is an area in need of more research.

In this study, our approach to math walks draws on the successful characteristics of informal math learning, as well as on place-based education, where local communities are sites and resources for learning, and active engagement in the community is facilitated (Sobel, 2004). Math walks are activities where learners visit a series of different locations, physically or virtually, and observe and ask questions about how math appears in their surroundings. Our approach to math walks leverages the pedagogical strategy of *problem-posing*, where learners ask and solve their own mathematical questions. In the math walks program, youth experience mathematics in their surroundings (e.g., homes, communities, and school settings) and create math walk stops based on their observations of their surroundings. The math walk stops youth created consist of the math problems students posed and the corresponding solutions.

One challenge of designing informal learning environments was that some individuals could feel uncomfortable knowing that mathematics was involved in the environment, and they were expected to connect the environment with mathematical topics (Gyllenhaal, 2006). By leveraging the problem-posing strategy, individuals can choose the topics to pose questions about and embed their prior knowledge, interest, and social and cultural background into the problems. As a result, the problem-posing strategy can alleviate individuals' anxiety about learning mathematics during math walks and help individuals develop more positive dispositions toward mathematics (Fetterly, 2010). Mathematical dispositions refer to the attitude to see mathematics as something logical, useful, and worthwhile (National Research Council., 2001). However, the *combination* of problem-posing and informal mathematics learning has received very little attention in the research literature.

Problem-posing has been described as referring “to both the generation of new problems and the re-formulation, of given problems. Thus, posing can occur before, during, or after the solution of a problem” (Silver, 1994; p. 19). This broad definition makes it difficult for educators to learn about what a problem-posing activity should look like, how to implement problem-posing activities, and how to scaffold their students during problem-posing. Even though a positive relationship between problem-posing and students' mathematics learning has been documented, a gap between research findings in problem-posing and actual implementation remains (Cai et al., 2015). In addition, very few studies have looked at problem-posing in *informal* learning environments, even though problem-posing is an ideal approach in contexts where students do not need to follow a prescribed curriculum or standards and are free to generate a wide range of mathematical ideas and connections.

To contribute to the extant literature on problem-posing and bridge this gap between problem-posing's implementation in creating informal learning environments, this study investigated youth's problem-posing performance and procedure in a math walk program called “walkSTEM.” It analyzed how this experience shaped students' dispositions toward mathematics. This study also aimed to look into youth's interactions with their peers and instructors by observing and analyzing their discussions and conversations when posing and solving math walks problems collaboratively. walkSTEM is an initiative in a large metropolitan area where youth, classes, and families take walks and find mathematical concepts and principles in the architecture, designed objects, art, and nature around them. When youth are tasked with creating their own math walks, they design “stops” on a math walk around their homes, communities, or schools, often leading their audience on the walk and explaining how mathematics is integrated into the surroundings. Since this study occurred during the COVID-19 pandemic, the math walks program that was implemented during a weekend extracurricular program for high school students was modified to be fully online. Youth met virtually with the instructors and other program members to watch existing math walk videos from their local communities and design their own walks collaboratively. In terms of their self-generated walks, youth can create walks around not only math topics but also other STEM topics. Even though most of the walks and the self-generated questions were related to mathematical topics, some youth in this program created questions related to biology, environmental science, statistics, and so on. As the objective of this program was to encourage students to connect their school-learned topics to real-world scenarios, the authors did not limit the topics to youth's self-generated walks. Given that remote learning has become more prevalent, this study explored the possibility of online math walks. It investigated both the advantages and challenges of implementing problem-posing and math walks through virtual formats.

The purpose of this study was to (a) investigate the problem-posing program's effects on youth's mathematical dispositions; (b) compare youth's problem complexity in different problem-posing tasks; and (c) explore the kinds of interactions youth have when creating math walks.

## 2. Theoretical framework

### 2.1. Problem-posing

Problem-posing “is a feature of broad-based, inquiry-oriented approaches to education” (Silver, 1994, p.21). Problem-posing has been an increasingly important research area in mathematics education in recent decades both in the United States (English, 1997; Walkington, 2017; Walkington and Hayata, 2017) and in other countries including China (Li and Lü, 2004; Chen et al., 2007), Singapore (Cai, 2003), Indonesia (Suarsana et al., 2019), and Turkey (Salman, 2012; Ozdemir and Sahal, 2018). Researchers also conducted cross-national studies on problem-posing to explore the mathematical achievement differences between students of different countries (Cai, 1998; Cai and Hwang, 2002; Cai and Jiang, 2017).

Extant studies suggested that integrating problem-posing in students' mathematical learning can positively impact students' problem-solving skills, problem-posing skills, conceptual understanding, and dispositions toward mathematics (Brown and Walter, 1990; Silver, 1994; Silver and Cai, 1996; English, 1997; Cai, 1998; Cai and Hwang, 2002; Singer et al., 2013; Kapur, 2015; Walkington, 2017). Wang et al. (2022) conducted a meta-analysis on mathematical problem-posing interventions from 21 studies and concluded that the estimated average effect size of problem-posing on students' mathematical learning outcomes was 0.64 *SD*. The mathematical learning outcomes analyzed included problem-solving skills, problem-posing skills, mathematical dispositions, and mathematical achievement.

### 2.2. Metacognitive skills and mathematical dispositions

Problem-posing activities can promote both students' metacognitive skills (Karnain et al., 2014) and their mathematical dispositions (Silver, 1994; Wang et al., 2021). Specifically, suppose students are given a mathematical problem, they are required to generate some similar problems. Students need first to analyze the problem holistically (Silver, 1994) and understand the dynamics of the given problem (Priest, 2009) before they start to generate their problems. After posing the problems, students also need to develop a more thorough understanding of the logical relations among the problem texts, the question sentences, and the solutions to the problems they posed (English, 1997; Cai, 1998; Priest, 2009). During these processes, students may constantly self-monitor and self-regulate, thereby improving their metacognitive skills. Baumanns and Rott (2022) investigated the individuals' problem-posing process and identified these problem-posing-specific metacognitive behaviors: planning, monitoring and control, and evaluating. Research has also discussed how students' engagement with problem-posing could stimulate students' interest in mathematics learning and reduce students' mathematics anxiety, which includes fear and avoidance of learning mathematics (Brown and Walter, 1990; Silver, 1994). Given the various formats of problem-posing tasks, Stoyanova (1999) categorized problem-posing into three types: free, semi-structured, and structured problem-posing. In structured problem-posing tasks, students re-formulated given problems or generated problems based on a specific solution. In semi-structured problem-posing tasks, students generated problems based on a given problem structure or solution structure. In free problem-posing tasks, there is no specification of which type of problem to pose or which area the problem should be based on.

In extant literature on problem-posing, researchers also analyze the complexity of student-generated problems to investigate the relationships among students' problem-posing performance, problem-solving performance, mathematical achievement, and the type of learning tasks students are engaged in. Silver and Cai (1996) analyzed the mathematical solvability, linguistic complexity, and mathematical complexity of students' posed problems. The linguistic complexity was coded with the number of assignment, relational, and conditional propositions presented in the student-generated problems. The mathematical complexity focused on the number of mathematical semantic structural relations (i.e., change, group, compare, restate, and vary) in the problems. One example the authors provided was Did Arturo drive a longer time than Jerome and Elliot drove altogether in a regular way? This problem included five semantic relations: compare, restate, group, restate, and vary. In this study, the authors assessed 509 middle school students' problem-solving and problem-posing skills. The problem-posing task was a word problem statement without a given question. Students were asked to pose three different questions that could be answered with the information in the provided statement. The results suggested that stronger problem-solvers also tended to pose more complex mathematical problems than their peers who were not as strong in problem-solving. English (1997, 1998) coded the complexity of children-generated problems by coding problem type and the whether the problems required multiple steps to solve. English (1998) also compared the complexity of children-generated problems in formal (i.e., standard symbolic addition and subtraction sentences) and informal contexts (i.e., a large photograph of children playing with brightly colored items) and suggested that children posed more diverse and complex problems in informal contexts than formal contexts.

### 2.3. Scaffolding strategies for problem-posing

Unlike other learning activities, most students do not have prior experience with problem-posing. Therefore, it is important to provide students with peer support and a learning environment within which they are motivated to raise various questions. Most student-centered active-learning strategies, such as inquiry-based learning, problem-based learning, and discovery learning, can help to create such learning environments (Albanese and Mitchell, 1993; Bicknell-Holmes and Hoffman, 2000; Hattie and Yates, 2009). In these student-centered learning environments, students can learn at their own pace, take on active roles to create and synthesize their own questions and knowledge, and make connections to real-world issues (Barron et al., 1998; Bicknell-Holmes and Hoffman, 2000). In addition, utilizing appropriate scaffolding strategies can enhance students' problem-posing experience. Peer interaction is one of the most prevalent scaffolding strategies for problem-posing (Gade and Blomqvist, 2015). Kontorovich et al. (2012) proposed a framework to analyze students' problem-posing process that includes five aspects: task organization, knowledge base, problem-posing heuristics and schemes, group dynamics and interactions, and individual considerations of aptness. Group dynamics and interactions refer to the processes of social nature that occur when a group work on a problem-posing task together is included in the framework. The authors demonstrated the usefulness of this framework by using it to explain the different reactions students had when engaged in problem-posing activities, despite the similar background these students shared. The authors suggested that this framework could be used to do a fine-grained analysis of student's problem-posing work and could account for hidden mechanisms involved in students' decision-making when creating their own problems.

We previously conducted a pilot study that investigated young children's participation in a walkSTEM afterschool program where they were asked to pose problems (Wang et al., 2021). The findings suggested that children were able to create meaningful and interesting problems based on their observations of the school buildings and playground. Children were engaged in group activities during the math walks program: they experienced math walks created by previous students and posed more problems about the contexts; they walked around their campus and asked questions in groups; they voted for the places they were most interested in to create math walk stops at; they solved their self-generated problems with group members, and they created a final video to showcase their math walk to their friends and parents. During this process, children participated in free problem-posing first to get to know the concept of creating their own problems, followed by doing semi-structured problem-posing that modeled good problem-posing products, and then back to doing free problem-posing and creating problems about their school and communities. This sequence seemed especially effective in scaffolding children's problem-posing work. A recent meta-analysis on problem-posing (Wang et al., 2022) also compared how the different types of problem-posing activities could affect students' mathematical learning outcomes and concluded that implementing a combination of free, semi-structured, and structured problem-posing was more effective than only implementing semi-structured or structured problem-posing activities. In addition, the pilot study findings also indicated that children became more positive about learning mathematics and became more independent learners after attending the program. However, whether a similar dynamic could be facilitated in an *online* context with older students was not clear. That study also involved just 10 students who were in a school setting working with their math teachers. Thus we set out to follow this investigation with a new study investigating problem-posing with math walks in an online extracurricular program for high school students.

## 3. Materials and methods

This study employed a mixed-method research design (Creswell and Clark, 2017) to investigate problem-posing activities' effects on mathematical dispositions and the problem-posing performance of youth. This section presents the research questions, the research methodology, and the activities included in the online math walks program.

### 3.1. Research questions

This study aimed to utilize the mixed-research design to comprehensively analyze youth's learning process and dispositions in this online math walks problem-posing program with qualitative and quantitative analyses. With the quantitative analysis, this study examined the trajectories of problem-posing performance throughout the program and compared dispositions toward mathematics before and after the program. With the qualitative analysis, the authors analyzed problem-posing work throughout the program and youth's interviews to further analyze how problem-posing shapes youth's mathematical interests and dispositions and what interactions occur among youth when they pose problems and create their own math walks. The research questions are as follows:

*(1) How does designing and leading a math walk shape youth dispositions toward math and toward creating their own math problems?*
*(2) How does the complexity of the mathematical problems students generate as part of their math walk activities vary over the course of the program?*
*(3) What interactions do youth have with their peers when they pose problems and design their math walk questions and stops?*

### 3.2. Methods

#### 3.2.1. Participants

Participants were recruited from an existing extracurricular college preparation program in a university located in a large southwest metropolitan area. The program's objective is to help first-generation students from designated schools who desire to pursue college transition from high school to college. Activities were enacted during Saturday morning sessions. The program accepted students from 10 schools, where 76.45% of the students are economically disadvantaged, and 24.38% are English learners.

In total, 35 students were recruited (26 Hispanic, seven African American, one Asian, and one student who identified as two or more races). Among the 35 students, there were 24 female and 11 male students. All participants were high school students, and there was one freshman, 13 sophomores, four juniors, and 17 seniors. The 13 instructors (11 females and two males) in this program were tutors in the college preparation program, who were all undergraduate students from this university. Of the 13 instructors, seven were Hispanic, three were White, two were Asian, and one was African American.

#### 3.2.2. Problem-posing activities in the online program

In the virtual math walks program, there were three main problem-posing activities for students: watching walkSTEM videos and posing their own problems based on those videos, taking #STEMlens photos and posing problems based on those photos, and creating virtual math walks and presenting the walk in small groups.

The walkSTEM videos were short videos in which prior youth or informal STEM educators discussed STEM-related problems in their surroundings. The STEM problems could be based on a place (e.g., a museum, a shopping mall, and a park), an activity (e.g., playing basketball and playing music), or a STEM topic or concept (e.g., geometry and biology). After watching the videos, students were asked to complete a video-watching questionnaire (see Appendix A). Students documented the questions being asked in the video, explained how the video was related to mathematics, and created problems about the scene or the object in the video. The #STEMlens photo was a problem-posing activity in which students took photos of their surroundings, marked up the photos using photo-editing tools, and posed problems based on the photo and markups. Students' #STEMlens photos were assessed by their instructors using the rubric presented in Appendix B2. Creating a STEM walk was the final project of the program. Each student designed three walk stops, and each stop was comprised of a #STEMlens photo or a short video, a STEM question about the photo/video that students posed, and a corresponding answer or a strategy to answer the question. Students worked in groups to provide feedback and suggestions to each other. Each selected one stop from their STEM walk and presented in groups to their peers, parents, staff, and instructors. The project and the presentation were scored by their instructors using the rubrics in Appendix B1. Among these three activities, the problem-posing work in the video-watching activity would be considered semi-structured problem-posing, according to Stoyanova (1999), as students were asked to create problems based on a given picture or scene. On the other hand, the problem-posing in #STEMlens and the Final Walk project would be categorized as free problem-posing. Students were allowed to pose problems based on objects in their own surroundings.

Students met with their instructors nine times for the program during the semester, including three longer sessions (one 90-min session and two 120-min sessions), five 30-min check-in sessions, and one final presentation session. The researchers, the program coordinators, and the college preparation program staff met with the instructors for training purposes before implementing the program. More descriptions of the instructional activities in each session are listed in Table 1, and the researcher provided detailed lesson plans for all sessions to instructors before each session.

**Table 1:** Student activities in each math walk session.

**Session**	**Math walks program activities**
Session #1	Students completed the pre-survey. Instructors introduced the walkSTEM program, the gameboard, and the #STEMlens photos. Students watched one walkSTEM video and completed the video-watching form
Session #2	Students watched three walkSTEM videos and completed three video-watching forms. Instructors checked in with students regarding their #STEMlens photos
Session #3	Instructors checked in with students regarding their #STEMlens photos. Students submitted at least one #STEMlens photo. Students who finished earlier would watch two more walkSTEM videos and complete the forms
Session #4	Instructors introduced the Final Walk project to students by watching previous student-created Final Walk videos. Each student completed a Final Walk project planning sheet and started to work on the first two math walk stop design worksheets
Session #5	Students completed the first two math walk stop design worksheets and finalized at least one math walk stop, including the question, the photo/video, and the response to the question for the stop. Students who finished early would watch one more walkSTEM video and complete the form
Session #6	Students started to work on the third math walk stop design worksheet, watched one walkSTEM video, and completed the form
Session #7	Students worked in groups to each select one math walk stop from their projects to form a group Final Walk. Students gave feedback to each other, wrote the script for their Final Walk, and created the slides for the presentation on STEM day
Session #8	Students finalized their group's Final Walk presentation and rehearsed
Session #9	Students presented their group's Final Walk to their parent's peers. Students completed the post-survey after the presentation

#### 3.2.3. Measures

Research data were collected through six sources: the student pre- and post-survey, the instructor pre- and post-survey, the instructor mid- and post-interview, the student post-interview, the students' problem-posing work, and the video recordings of all of the meetings.

The students' pre- and post-surveys are presented in Appendix C. Students took the pre-survey during their first meeting, which included questions about demographic information, problem-posing, problem-solving, conceptual understanding, procedural fluency, and mathematical dispositions items. The student post-survey was implemented after the final presentation day, and the post-survey only included items on students' problem-posing skills and mathematical dispositions. The dispositions survey items were adapted from the mathematical individual interest scale from Linnenbrink-Garcia et al. (2010). Cronbach's alpha for the mathematical interest scale was 0.90, which indicates good reliability. The procedural fluency, conceptual understanding, and problem-solving items were selected from TIMSS 2011 grade 8 mathematics assessment (Mullis et al., 2012). The overall Cronbach's alpha for the TIMSS 2011 achievement scores was 0.97 (Bofah and Hannula, 2015).

Students who participated in all three problem-posing activities were selected to be interviewed using the interview protocol in Appendix D after their final presentations. The interview protocol focused on students' problem-posing experiences in the program, the difficulties or challenges in generating problems, and whether students' mathematics dispositions had changed after participating in this program.

#### 3.2.4. Coding and analysis

Student-generated problems' content complexity and students' ratings in the mathematical dispositions survey were the main quantitative outcome variables in this study. The content complexity was coded with the criteria adapted from Liu et al. (2020). The coding categories with examples and problem-posing prompts for the example problems are presented in Table 2. We coded student-created problems on a scale of 0–5, where 0 is the least complex and 5 is the most complex. We measured the complexity of the problem from three perspectives: whether the problem is relevant to the prompt, whether the problem statement is ambiguous or not, and whether the problem allows for multiple solutions. An example problem with a complexity rating of 5 is in Table 2: How does the color and space between each color make this picture pleasing to the eye? This is a non-routine problem that usually does not exist in a math textbook, and there are multiple perspectives and strategies to answer this question. For instance, we could measure the distance between each circle, calculate the portion each circle is covered, explore the different shapes created by the set of circles, and check the RGB information of the colors to understand if any of these factors make the picture pleasing to the eye. Cohen's kappa (Cohen, 1960) was utilized to calculate the reliability of the content complexity coding manual. Notably, 54 problems were selected randomly from a total of 140 problems in three separate sets to be double-coded by the researcher and a second rater. The weighted kappa was 0.81, which is considered a good agreement (Landis and Koch, 1977).

**Table 2:** Content complexity scoring examples.

**Category**	**Score**	**Examples**	**Problem-posing prompts**
Not-relevant or incomprehensible	0	All circles together. (Prompt A)	Prompt A 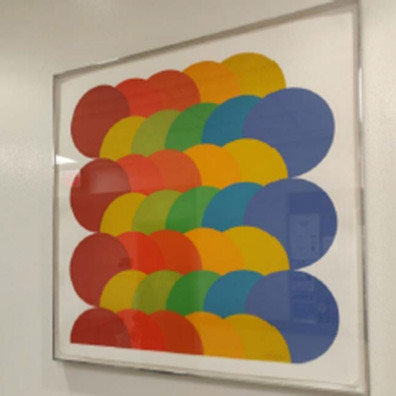 Prompt B 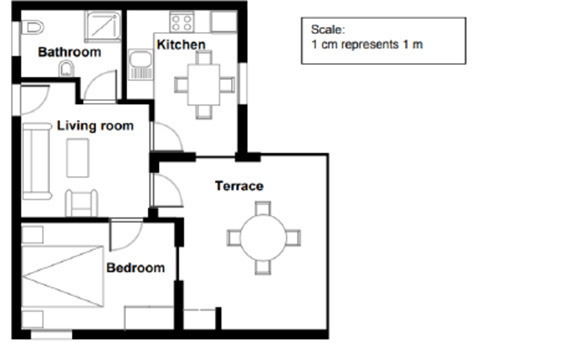 Pose a mathematical problem based on this apartment floor plan or this apartment
Relevant statement	1	This could be a probability question. (Prompt A)	
Relevant problem, but with ambiguity	2	Why were they built like that? (Prompt B)	
Relevant problem without any ambiguity	3	From just looking at the picture, how many circles can be calculated by each color? (Prompt A)	
Non-routine relevant problem without any ambiguity	4	If the real estate agency wanted to renovate and deduct 10 meters in the living room to give more space to both Terrace and kitchen, what would be the area of the Living room? (Prompt B)	
Non-routine relevant problem without any ambiguity; problem allows for multiple solutions	5	How do the color and space between each color make this picture pleasing to the eye? (Prompt A)	

We compared students' *mathematical dispositions* with their responses in the pre- and post-mathematical disposition surveys with a paired *t*-test. In total, there were 17 students who finished both the pre- and post-surveys (35 pre-survey, 18 post-survey). Next, a linear mixed-effects regression model was used to compare the *content complexity* of student-generated problems in different problem-posing activities. The model was fit with student ID as a random effect. Student characteristics (i.e., the pre-survey math interest, pre-test procedural fluency score, pre-test conceptual understanding score, pre-test problem-solving score, gender, and grade level) were tested for significance as covariates. The three problem-posing activities during the math walks program were also included in the model, along with the pre- and post-survey problem-posing tasks as covariates. In this model, each data point was one student creating one problem. In total, there were 261 student-created problems, including 134 video-watching activity problems, 44 #STEMlens photo problems, 30 Final Walk problems, 35 pre-survey problem-posing task problems, and 18 post-survey problem-posing task problems.

The linear mixed-effects model was fit using the linear mixed-effects regression (*lmer*) command from the *lme4* library in R (Bates et al., 2015; R Core Team, 2016). The mixed-effects model was selected as it allowed us to use all the data despite students completing different numbers and types of problem-posing tasks. It could also account for the partially clustered data.

The qualitative analysis portion of this study employed a single-case-study design (Creswell, 2013). The identified case in this study was the math walks program at the college preparation program. Thematic analysis was employed to identify and examine themes that emerged from the data following the six-phase procedure presented in Braun and Clarke (2006): familiarizing yourself with your data, generating initial codes, searching for themes, reviewing themes, defining and naming themes, and producing the report. In light of the findings in the pilot study described earlier, some potential coding foci that the researcher paid particular attention to are listed in Appendix E.

## 4. Results

Table 3 presents descriptive statistics for the measures. Due to the online format of this program and its implementation toward the beginning of the COVID-19 pandemic, the attrition rate was fairly high. There were 35 pre-survey responses and 17 post-responses. To understand if students who left the program were different from students who finished, the authors conducted an independent *t*-test on these two groups' pre-survey interest and pre-survey problem-posing complexity. The independent *t*-test result revealed that the difference in students' pre-survey dispositions was not statistically significant, *t* (32) = −0.23, 95% CI = [−0.54, 0.43], *p* = 0.82. However, the difference in students' pre-survey problem complexity was statistically significant, *t* (34) = 3.67, 95% CI = [0.69, 2.39], *p* < 0.001. In other words, there was not enough evidence that students who dropped off from the program had more positive or negative dispositions toward mathematics. However, students who stayed in the program were able to pose more complex problems from the beginning of the program than their counterparts.

**Table 3:** Descriptive statistics of all measures.

**Variable name**	***n***	***M***	**SD**
Pre-survey interest in mathematics	35	3.63	0.75
Post-survey interest in mathematics	18	3.88	0.64
Pre-survey posed problem content complexity	31	2.77	1.15
Post-survey posed problem content complexity	16	3.41	1.08
Video-based problems content complexity	18	3.13	0.20
#STEMlens content complexity	15	3.15	0.39
Final walk content complexity	12	3.83	0.33
Pre-test procedural fluency score	35	2.73	1.12
Pre-test conceptual understanding score	35	2.84	0.89
Pre-test problem-solving score	35	1.39	1.24

The average complexity of student-generated problems in the pre- and post-survey and the different problem-posing learning tasks are included in rows 3–7 of Table 3. The data suggested that the average complexity of student-generated problems for the Final Walk was higher than the other two learning activities in the program (#STEMLens photos and walkSTEM videos). The average complexity of student-generated problems in the post-survey is also higher than in the pre-survey.

### 4.1. RQ1: Students' dispositions toward mathematics and problem-posing

The Shapiro-Wilk's test for the difference between pre-survey and post-survey interest mean indicated that the difference was normally distributed (*p* = 0.91; Shapiro and Wilk, 1965). The test of homogeneity of variances indicated that the variances were not significantly different from each other, *F* (1.32) = 0.15, *p* = 0.70. The paired *t*-test result revealed that the improvement in students' interest from pre-survey to post-survey, 0.15, 95% CI [−0.10, 0.41], was *not* statistically significant, *t* (16) = 1.28, *p* = 0.22.

Following the quantitative analyses, we used thematic analysis to analyze the transcripts of the post-intervention student interviews, and the following themes emerged from the analysis.

Eight out of the 10 students being interviewed mentioned that they started to think more deeply and positively about mathematical concepts. One student (female, grade 10) explained as follows:

[the program] actually gives you a reflection of yourself that you did not know. Because something as a student you just ask like, why would the teacher ask me this kind of question. And when you do this kind of project you actually understand what situation the teacher was in and why did she ask this question. … In this kind of program, I think you'll actually understand and have more, more understanding, and more clarification on questions.

**Figure 1:**
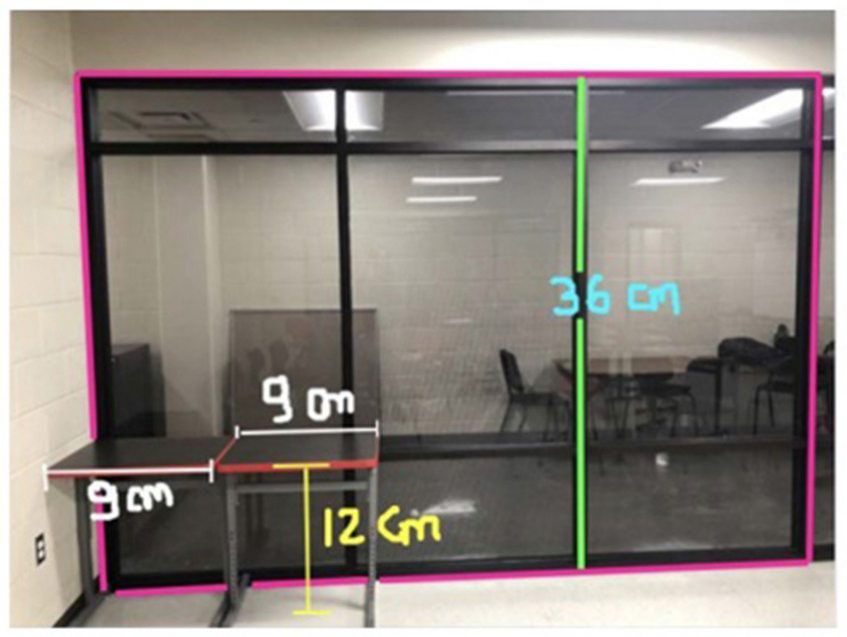
#STEMlens activity student work—The window. 1-How many tables do we need to fill the whole window? 2-How many rows are we going to create? 3-How many columns are we going to create?

The same student also described her experience with the #STEMlens photo activity to further demonstrate a similar idea. The picture and questions she mentioned are presented in Figure 1.

So one of the picture I took was the picture of my window. So I think, I like the creativity because when you create the question sometimes can't get that type of question… But I have multiple questions, I have other things we can actually put on the thing that were kind of complicated. So I was proud of myself because that makes me think I still remember I still have that kind of … the capacity, memory, how you can interpret real-life problems … I found myself asking questions that the teacher doesn't even ask.

Five students expressed that they became more interested in mathematics to some extent. One female student in grade 12 stated:

Just slightly more it's not like I really got into math or I really got into science but I really like it increased my like interest on it. Just to think about like why doesn't it happen or how is this related with stuff that I've learned before but I've never paying attention to it.

Three students mentioned that they were more patient and perseverant when solving mathematical problems after the intervention. In this program, students were only required to solve their self-generated problems in the Final Walk project, and students' Final Walk problems were the most complex according to the coding manual. That is to say, students spontaneously chose to pose and solve problems that were more complex and required more effort to answer. Students described the problem-solving process here as research and highlighted that it was different from the textbook problems they were used to

It was a good experience and then I get I got to learn more about it how it really is to do a research most importantly because I think it's good … it help me like think more about how they kind of research really goes and I mean, it's not a full research. It's not a full research but I got like a glimpse of it (female, grade 12).

Yes, Because I think I learned more I gain more experience on how to solve stuff, having patience, because it can be hard at some point, but having patience, take it easy … we can find a solution (female, grade 12).

Thus, the quantitative and qualitative results were not consistent.

### 4.2. RQ2: The complexity of students' posed problems

**Figure 2:**
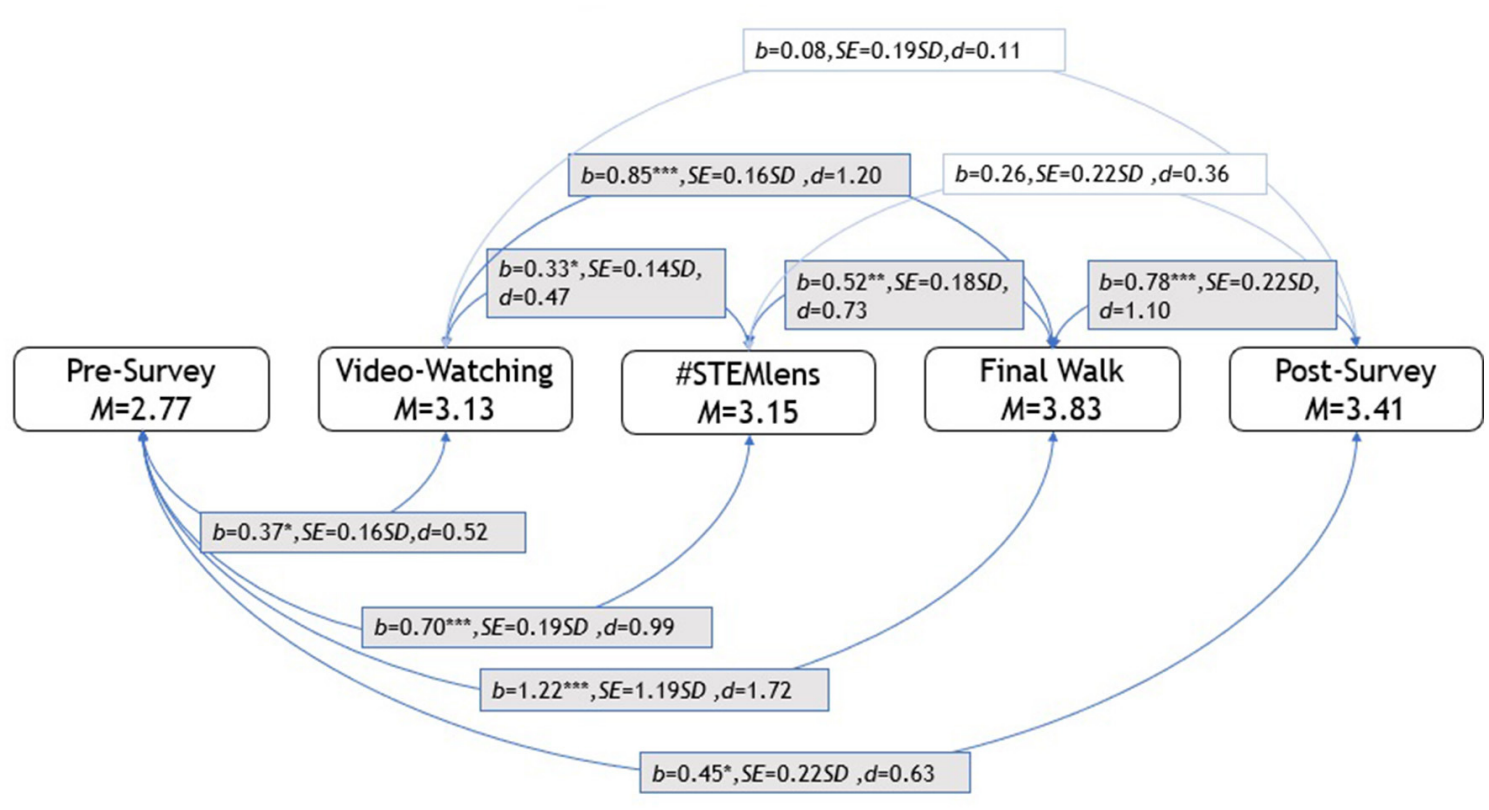
Pairwise comparison results of student—Generated problems.

The mixed-effects model was employed, and the regression results and Cohen's *d*-effect sizes are presented in Table 4. The effect sizes were calculated from the estimated marginal means with the estimated marginal means, aka least-squares means (*emmeans*) package in R (Russell, 2023). The regression results suggested that students' post-survey problems were more complex than pre-survey problems (*b* = 0.45, *p* = 0.047, *d* = 0.63). The results revealed that the Final Walk problems' complexity was significantly higher than all other problems. Final Walk problems were more complex than #STEMlens (*b* = −0.52, *p* = 0.0006, *d* = −0.73) video watching (*b* = −0.85, *p* < 0.0001, *d* = −1.20), post-survey (*b* = −0.78, *p* = 0.0004, *d* = −1.10), and pre-survey problems (*b* = −1.22, *p* < 0.0001, *d* = −1.72). On the other hand, the pre-survey problem complexity was significantly lower than all other problem complexities. In addition, the video-watching problems were less complex than the #STEMLens problems (*b* = −0.33, *p* = 0.017, *d* = −0.47). As introduced earlier, the Final Walk and #STEMlens activities were categorized as free problem-posing, and the video watching was considered semi-structured problem-posing, according to Stoyanova (1999). The results showed that students posed more complex problems in free problem-posing activities (i.e., Final Walk, #STEMlens) than in semi-structured problem-posing activities (i.e., video watching). All pairwise comparison results and corresponding effect sizes are presented in Figure 2.

**Table 4:** Mixed-effects linear regression model comparing problems' complexity—Pre-survey problem-posing task as reference group (No. of observations: 261).

**Random effect**	**Variance**		**SD**			
Student ID	0.44		0.66			
Fixed effects	*B*	*d* ^a^	*SE*	95%CI	*p*-value	Sig.
(Intercept)	0.97		1.41	[−1.80, 3.74]	0.50	
Pre-survey problem-posing task	(ref.)					
#STEMlens photo	0.70	0.99	0.19	[0.33, 1.08]	0.002	**
Final walk project	1.22	1.72	0.19	[0.85, 1.60]	< 0.0001	***
Video-watching activity	0.37	0.52	0.16	[0.05, 0.69]	0.02	*
Post-survey problem-posing task	0.45	0.63	0.22	[0.006, 0.89]	0.048	*
Pre-survey math interest	0.06		0.26	[−0.46, 0.57]	0.83	
Pre-test procedural fluency score	0.11		0.16	[−0.20, 0.43]	0.50	
Pre-test conceptual understanding score	0.33		0.23	[−0.12, 0.79]	0.17	
Pre-test problem-solving score	0.04		0.18	[−0.30, 0.39]	0.79	
Gender female	(ref.)					
Gender male	−0.68	−0.96	0.33	[−1.33, −0.04]	0.05	*
9th Grade	(ref.)					
10th Grade	0.33	0.46	0.77	[−1.17, 1.83]	0.68	
11th Grade	−0.13	−0.19	0.84	[−1.77, 1.51]	0.88	
12th Grade	−0.03	−0.04	0.73	[−1.47, 1.41]	0.97	

Adjusted *R*^2^ = 0.58, RMSE = 0.67.

a Cohen' *d* effect sizes are calculated with the *emmeans* package through estimated marginal means (Russell, 2023).

^*^indicates the correlation is significant at the.05 level (two-tailed), *p* < 0.05.

^**^indicates the correlation is significant at the.01 level (two-tailed), *p* < 0.01.

^***^indicates the correlation is significant at the.001 level (two-tailed), *p* < 0.001.

One student's problem-posing work is presented in Table 5 to show the problems at different stages throughout the program. Eric was a 10th grader in the program with a pre-survey mathematical interest rating of 2.75 on a 5-point scale. Eric watched 14 walkSTEM videos and submitted 19 #STEMlens photos. We listed five video-watching problems, five #STEMlens problems, the Final Walk problems, and the pre- and post-survey problems that Eric posed in the table. The problems Eric created for the #STEMlens activity showed that he was able to pose more and more complex and creative problems about his surroundings. For example, #STEMlens #1, #2, #10, and #13 were all about geometry concepts and measurements. The first two problems were similar to textbook problems students were accustomed to solving and were less creative. However, the #10 and #13 problems did not directly ask for a measurement but focused on how the shape of the chip container could affect the volume and how the positions of the fan blades could affect the efficiency. In addition, another theme that emerged from his #STEMlens submissions was the number of photos and problems he was able to create in the same environment. Eric took 5 #STEMlens photos and created accompanying problems in his backyard, which demonstrated how he was able to see various STEM topics and problems in the surroundings.

**Table 5:** Eric's problem-posing work.

**Activity**	**Problem-posing**	
Pre-survey	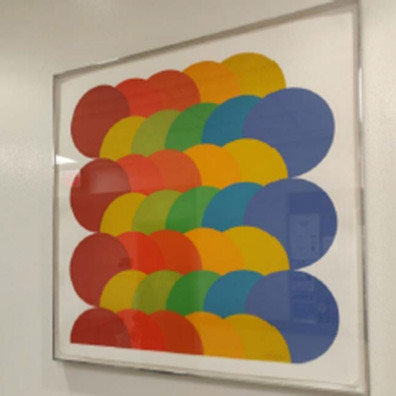	From just looking at the picture, how many circles can be calculated by each color? What is the length of the bathroom and kitchen different from the length of the bedroom to the terrace by millimeters?
	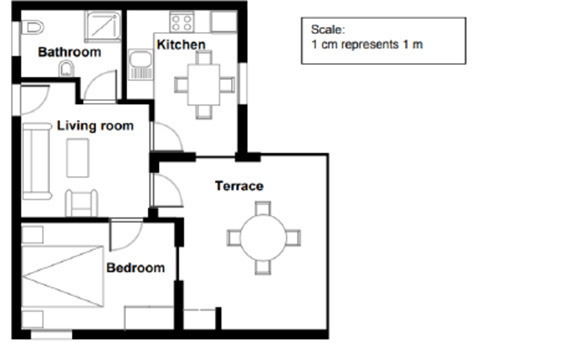 Pose a mathematical problem based on this apartment floor plan or this apartment	What type of measurement is used to determine that each part is equal? If I were to be on the other side of the globe and someone else was on the opposite side, would the time be the same?
Video-watching	talkSTEM Videos: https://youtu.be/5GCxIvRpKSA https://youtu.be/vg5AZEP-ZcE https://youtu.be/SJ4QwU_xSlg	How many toppings can I add to my drink? If 200 cells can fit on a top of a pen, how many cells does it take to run a whole mile? That is one of many bridges in Dallas. Can the same math be added to another bridge?
#STEMlens	Student submitted 19 #STEMlens photos.	
	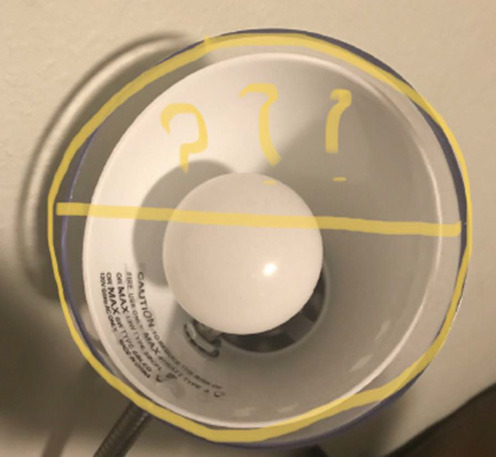	#1: What is the radius and/or the diameter of this lamp's circular form?
	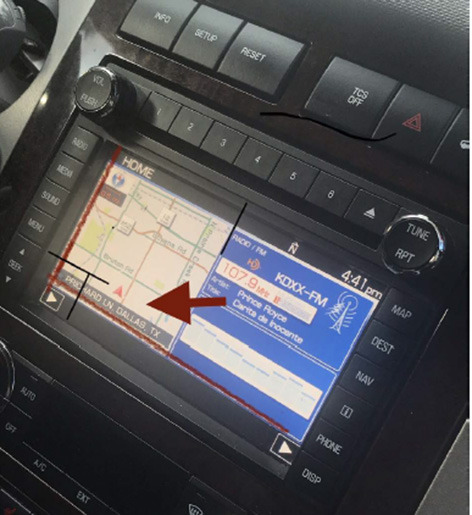	#2: What could be the area of the degree of the square-size tablet?
	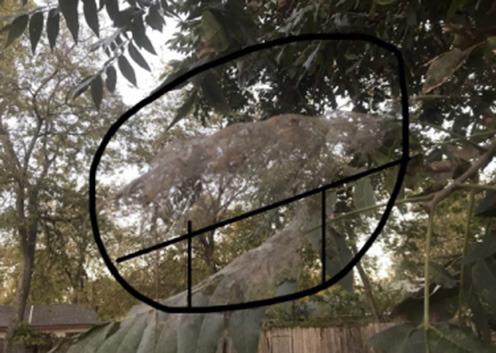	#6: In my backyard, there is a huge tree, bigger than my house, and I have noticed that the smaller branches are usually pulled down because of the spider webs. Question: Does the size of the spider's web really affect how the smaller branches are pulled? And is the spider's webbing good enough to catch prey?
	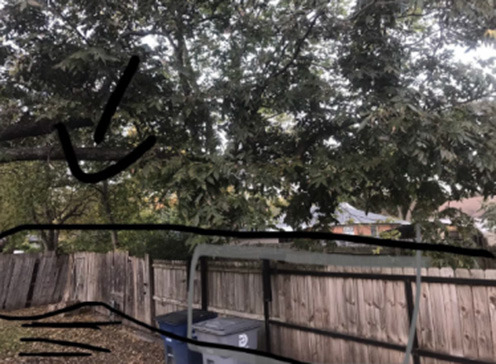	#8: From the picture, I have speculated that the wooden walls in the backyard are falling. Question: What would be the cause of the wood falling? Metal bars have been added to support it, but even so, they still fall. Is there a logical explanation for the wood getting weaker?
	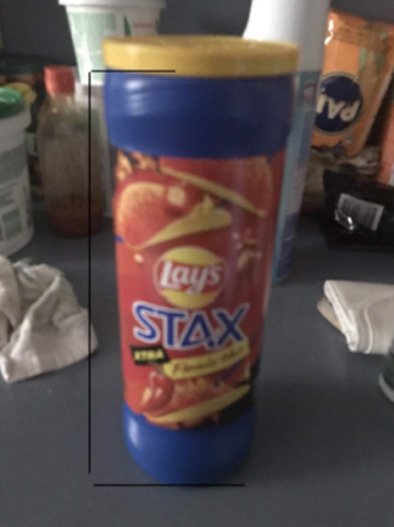	#10: Can the size of the bag or box affect the amount of chips inside it? Or, to be more specific, can you say a cylindrical shape holds more chips than a box or a bag?
	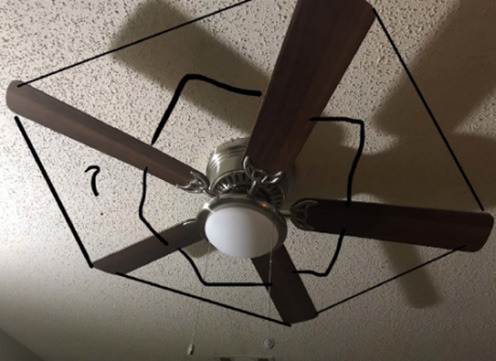	#13: Do the fans work more effectively if they are far apart from each other to a certain degree?
Final walk	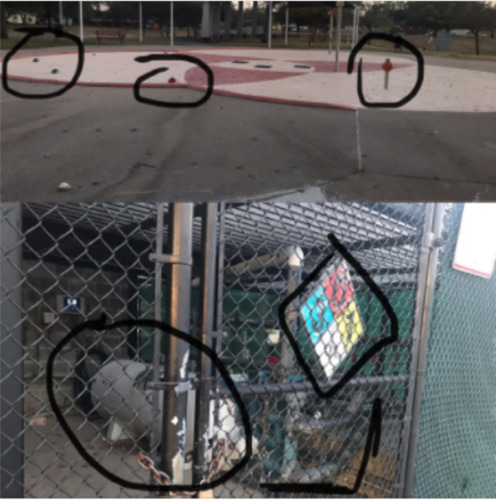 I wonder why there are so many things to power one small water park, and what intrigues me is how it is used; it is useful for sanitization and other reasons. How much water was possibly used daily? Also, from the sign shown, what kind of chemicals were added to the water and for what reason?	
Post-survey	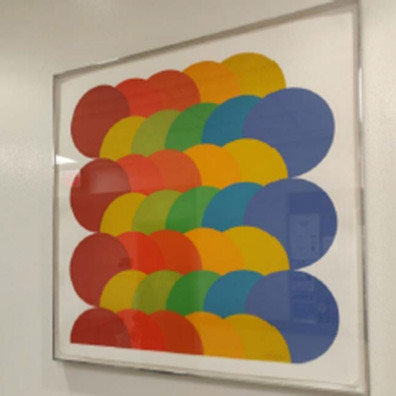	I see all of the circles on top of each other, and I would ask the question, What could the radius of all the circles be, and could they all be the same? I describe this picture as a way to figure out what the size of each circle could be. What could be the radius of each circle and are they all the same? From this picture, it makes me think about what could be the radius of each circle and which formula could help with that? And if each circle is the same size as each other
	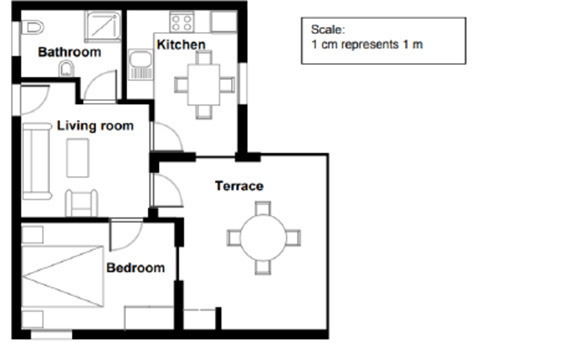 Pose a mathematical problem based on this apartment floor plan or this apartment	What could be the cm of each room of this house, and how you turn it into an m? What is the volume of the whole house by comparing each room's size? What could be the length of the whole house considering each room of the house?

### 4.3. RQ3: Students' interactions during the math walks program

We analyzed students' participation during the online meetings and identified one key type of interaction: students giving each other feedback and collaborating to create theme-based problems.

**Figure 3:**
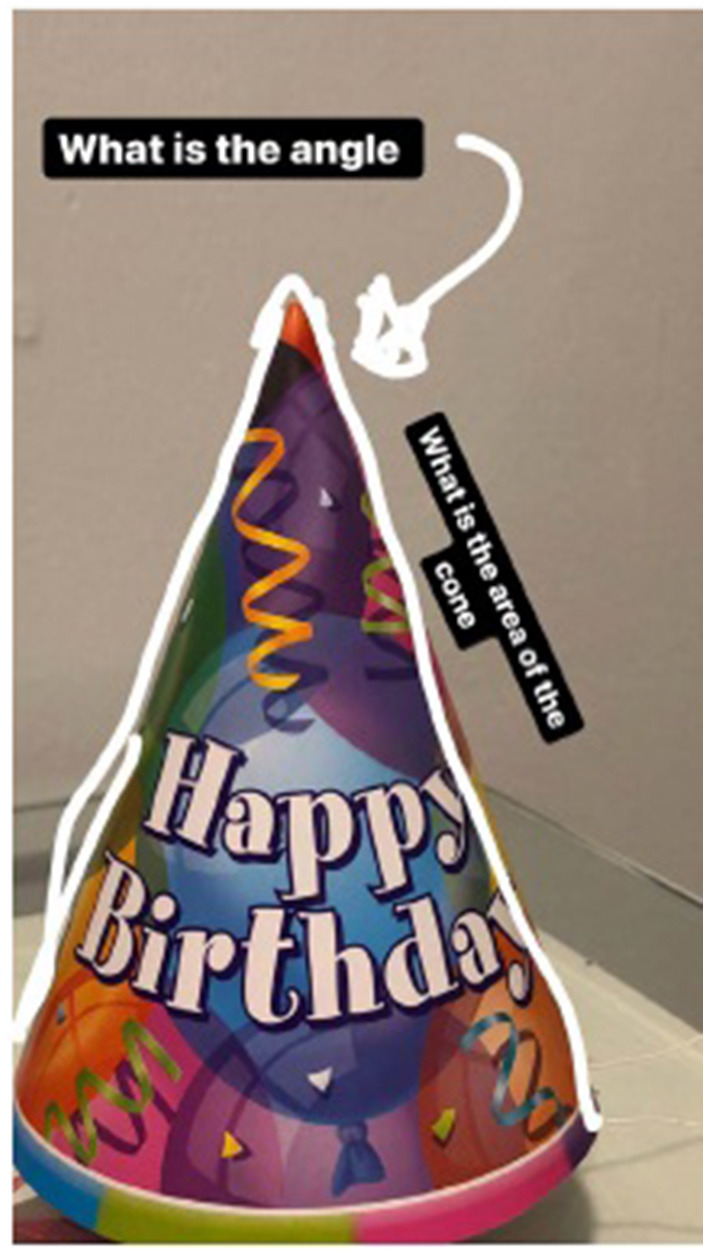
#STEMlens activity student work—The birthday hat.

In the #STEMlens and the Final Walk problem-posing activities, students were asked to pose problems based on the provided rubrics (Appendix D). The rubrics only talked about the quality of the photos and the markups, and the connection between the problems and the photos. In these two activities, students mainly worked independently except for when they were asked to evaluate each other's problems and provide feedback. Their feedback mostly only talked about the two aspects of the rubric. Below is an example of one student (male, grade 12) who talked about another student's #STEMlens (Figure 3) submission.

I will rate the question as a four I think. Because it is not that specific, it's just in the details. The markups, I think a four because you cannot see the complete image of the cone.

**Figure 4:**
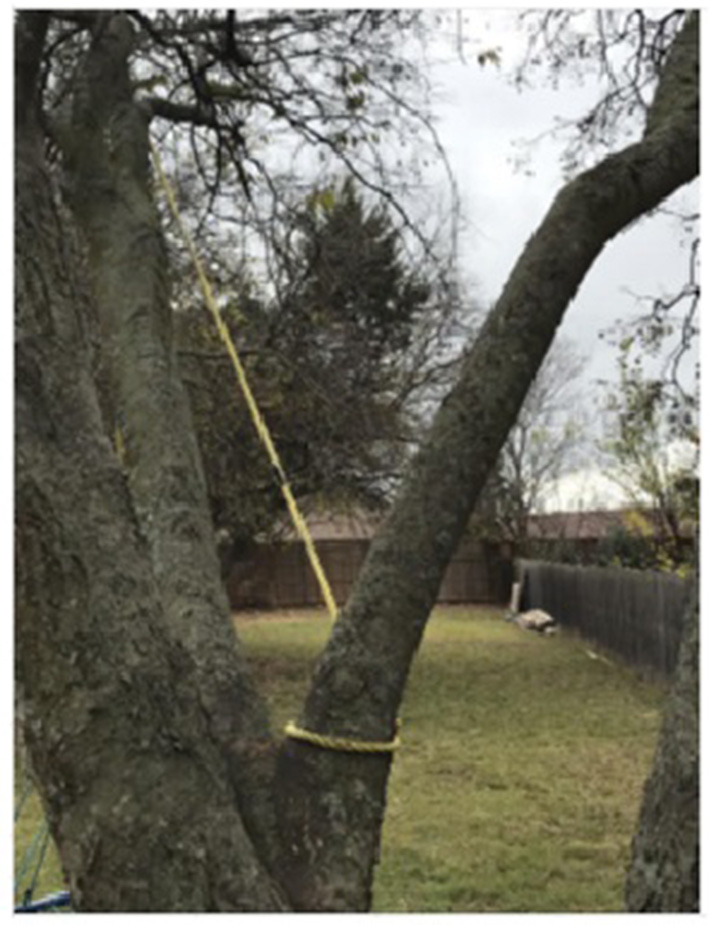
Abby's final work problem photo. Problem: what caused the tree to grow in that shape or form? Does it have to do with doil?

Once students became familiarized with problem-posing, they started to work on the Final Walk project. An added layer to this project compared to #STEMlens photos was the presence of a theme. Each group had to choose one theme, which could be a STEM topic, a place, or an interesting area. As a result, when students worked together in groups to create the Final Walk, they had to collaborate with each other to make sure their problems shared the same theme. In this excerpt, Abby (grade 12) started with a problem more related to geometry than biology, and she managed to modify her problem based on some feedback she received from Gina (grade 12) and the instructor. Abby's photo is presented in Figure 4. After this discussion, Abby modified here problem from “what is the space between the two branches” to “what caused the tree to grow in that shape or form? does it have to do with the soil?”

Abby: My photo was a tree like a tree branch in the form of a triangle. And I was going to ask, what is the space between both of the branches if I'm given a squared plus b squared equals c squared?

Instructor: So I guess my question to you is, would that be more related to biology or geometry with that question?

Abby: Geometry.

Instructor: Geometry, because you're talking about Pythagorean Theorem, a squared plus b squared plus c squared. So you kind of want to think about it in a more biological lens, if that makes sense. So other than Aurora, thank you for sharing, Jennifer and Nathalie. Anybody? What kind of questions can we ask about a tree that is in a that forms a triangle? What kind of questions we ask about it from a biological or environmental science lens, rather than a lens of geometry?

Gina: Maybe why the tree took that form? Like is there something else? Like if it got trapped between something or just why does it has that shape?

In this online program, students were not able to collaborate with each other in the same ways as they usually do in in-person meetings. Naturally, the peer collaboration rate decreased significantly as some students did not even turn on their cameras. However, once students started to work on the Final Walk project, they were more likely to critique each other's problems and discuss how they could pose different problems so that their problems could be integrated into a theme-based walk. In this online program, the Final Walk project was implemented last and fewer students participated in this Final Walk project than the #STEMlens activity due to the high attrition rate. However, instances in which students collaboratively pose problems only occurred during the Final Walk project. The two examples above showed how students interacted differently when evaluating their peers' problem-posing work in #STEMlens and the Final Walk project. In the first example, the student's comment only focused on the criteria in the #STEMlens rubric (e.g., the markup and the clearness of the photo). However, in the second excerpt, Gina proposed some new ideas and questions about the tree in Abby's photo, and Abby was able to connect her question to the group's theme (i.e., biology and environmental science) with Gina's suggestion.

## 5. Discussion

According to our quantitative analyses that investigated students' mathematical dispositions, there was not enough evidence to conclude that math walk activities enhanced dispositions. One explanation for this insignificant result is the small sample size. A recent meta-analysis calculated the average weighted effect size of students' dispositions after attending problem-posing interventions and reported an effect size of 0.54 (Wang et al., 2022). According to the power analysis with G^*^Power (Faul et al., 2009), in order to compare students' dispositions between two dependent means, the total sample size should be equal to or greater than 47. However, in this study, the sample size between pre-survey and post-survey mathematical disposition was 17, which made this analysis underpowered. On the other hand, the qualitative analyses revealed three themes related to how students were able to think differently and deeper about mathematical concepts, be more interested in mathematics, and be more perseverant in solving problems. However, these effects may not have shown up in the interests survey if students still saw math walks as being disconnected from “school math.”

As introduced earlier, students participated in both semi-structured and free problem-posing. The results suggested that students were able to pose more complex problems by the end of the program in the post-survey than in the pre-survey, which validated the positive effect of this online program. In addition, students posed more complex problems in the Final Walk project than in the video-watching activities and the pre- and post-survey, which resonated with the finding from the meta-analysis introduced earlier (Wang et al., 2022) that including free problem-posing tasks could increase students' performance. However, the results also indicated that even though both #STEMlens and Final Walk were free problem-posing tasks, the problems students generated in the #STEMlens activity were significantly less complicated than the Final Walk problems. The main difference between the #STEMlens and Final Walk project was the peer collaboration and the presentation. Students were able to collaborate as a group, review each other's problems, provide feedback, and solve the problems together in the Final Walk, which may have promoted more problem complexity.

In short, students tended to pose more complex problems in a free problem-posing task than in a semi-structured problem-posing task. Moreover, collaborating with peers to pose and solve problems and the requirement to present the problems to the audience also was associated with more complex problems. This result provides evidence for the authentic audience effect discussed in Crespo (2003): Introducing an authentic audience (e.g., sharing student-generated problems with others to solve) could motivate students' active participation in problem-posing.

### 5.1. Limitations and future directions

The limitations of this study were discussed from three perspectives. First, when generalizing the research findings to other students or other problem-posing interventions, caution should be taken. All of the meetings in this program were delivered through virtual online meetings. In addition, this program was implemented during a pandemic, and the majority of the students were already attending online classes all day from home. As a result, it could be difficult for students to be fully engaged in all of the activities and meetings, and the instructors were not able to monitor students' learning progress. Second, the small sample size was relatively small for quantitative analyses. As suggested above, these were the challenges and limitations caused by the online format and the special time of the program. The researchers employed this mixed-method research design and used various data sources to triangulate the findings and results to address this limitation. Finally, we acknowledge that our positionalities (as an international doctoral student and a faculty member interested in mathematics education and problem-posing) impact analyzing data and interpreting results and findings in this study.

This study tested and established the possibility of implementing a purely online math walks program. In prior studies, math walks were mostly implemented through in-person programs where children and youth meet with their facilitators at the learning sites (Lancaster, 2021; Wang et al., 2021; Martínez-Jiménez et al., 2022). This study provided future researchers with some insights about implementing a completely virtual math walks program. When designing and implementing online programs, future researchers should especially pay attention to developing collaborative activities to increase participant engagement and peer interaction levels. These collaborative activities are not only effective scaffolding strategies to support students' learning activities but can also potentially address the high attrition issue with online programs. These research findings also provide educators who are interested in implementing problem-posing with their students an easy-to-administer plan for afterschool programs or other informal learning environments. This study gives an idea of the kinds of interactions and problem characteristics to look for, as well as the ways in which such a program might effect or not effect outcomes that educators are interested in. Although this online program was implemented with high school students, the pilot study published by Wang et al. (2021) explored how a math walk could be administered to early elementary students. Hence, multiple different age ranges are possible. In addition, future research should investigate the students' performance in different types of math walks tasks on a large scale and explore how to use the different math walk tasks to develop a more student-friendly, personalized, and interactive program for youth. Moreover, in this study, the quantitative results on students' problem-posing indicated no significant difference in students' mathematical disposition. However, the qualitative analysis results revealed that students were able to think differently and deeper about mathematical concepts and became more interested in problem-posing. Hence, future researchers can employ more targeted measures, such as the attitudes toward problem-posing (ATPP) questionnaire from Nedaei et al. (2019), to better capture the change in students' dispositions toward problem-posing. In addition, some extant literature has investigated students' problem-posing performance by responding to different problem-posing prompts. Zhang et al. (2022) analyzed 669 elementary school students' problem-posing work and concluded that students performed better in problem-posing tasks with specific numerical information than in tasks without numerical information. Future research should investigate how different types of problem-posing prompts and programs can affect students' problem-posing work and behaviors. Finally, increasing levels of problem complexity seem to signal deeper thinking about mathematics but can be highly task specific. Future research should examine methods for having students pose authentic and community-imbedded problems.

## 6. Conclusion

This study employed a mixed-method research design to investigate an online math walks program's effects on students' mathematical dispositions and problem-posing performance. The online math walks program created an informal STEM learning environment for youth and engaged them in a series of problem-posing activities. The results partially validated how the math walk informal learning environment and the problem-posing activities youth participated in influenced youth to develop more positive mathematical learning dispositions. Through posing problems in their homes and communities, youth were able to think deeper and differently about mathematical concepts and make connections between school math and real-world applications. This study also compared youth's problem-posing work in different learning activities. It concluded that youth posed more complex problems in free problem-posing tasks when they were instructed to collaborate with each other to create problems and present their self-generated problems to the audience.

## Data availability statement

The raw data supporting the conclusions of this article will be made available by the authors, without undue reservation.

## Ethics statement

The studies involving human participants were reviewed and approved by Southern Methodist University Institutional Review Board. Written informed consent to participate in this study was provided by the participants' legal guardian/next of kin. Written informed consent was obtained from the individual(s), and minor(s)' legal guardian/next of kin, for the publication of any potentially identifiable images or data included in this article.

## Author contributions

Both authors listed have made a substantial, direct, and intellectual contribution to the work and approved it for publication.
