# Supplementary material for: Investigating problem-posing during math walks in informal learning spaces
Source: Front Psychol. 2023 Mar 6;14:1106676. doi: 10.3389/fpsyg.2023.1106676 (PMC10027002; doi:10.3389/fpsyg.2023.1106676)
Supplement: Supplementary file 1 [file Data_Sheet_1.docx]

# Appendix A: Video-Watching Questionnaire


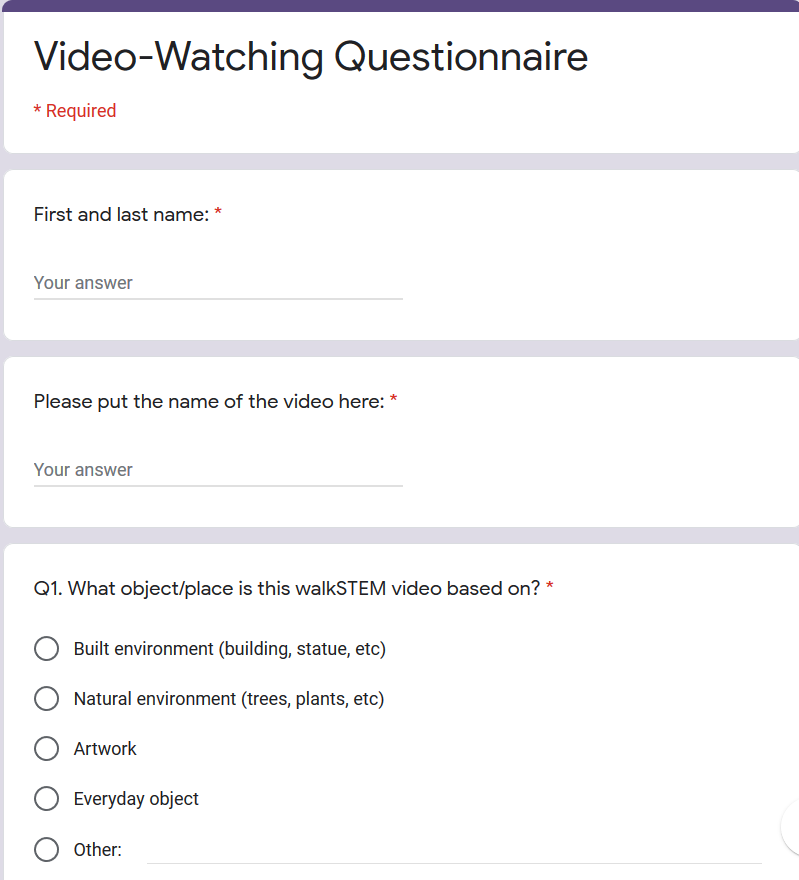

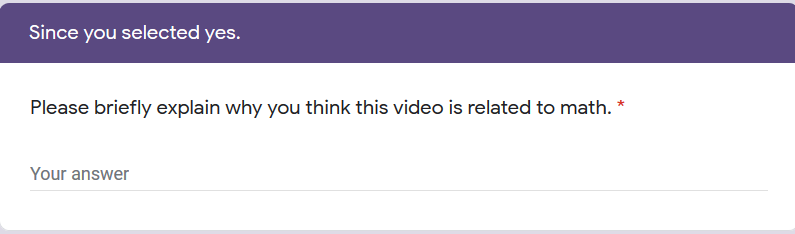

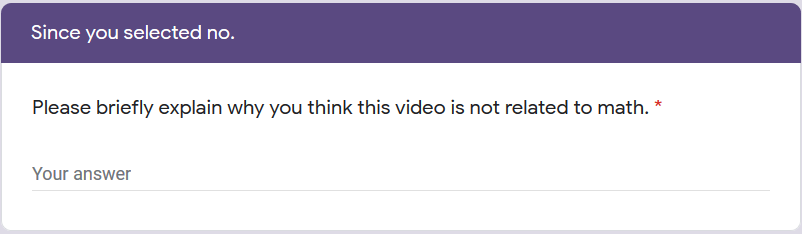

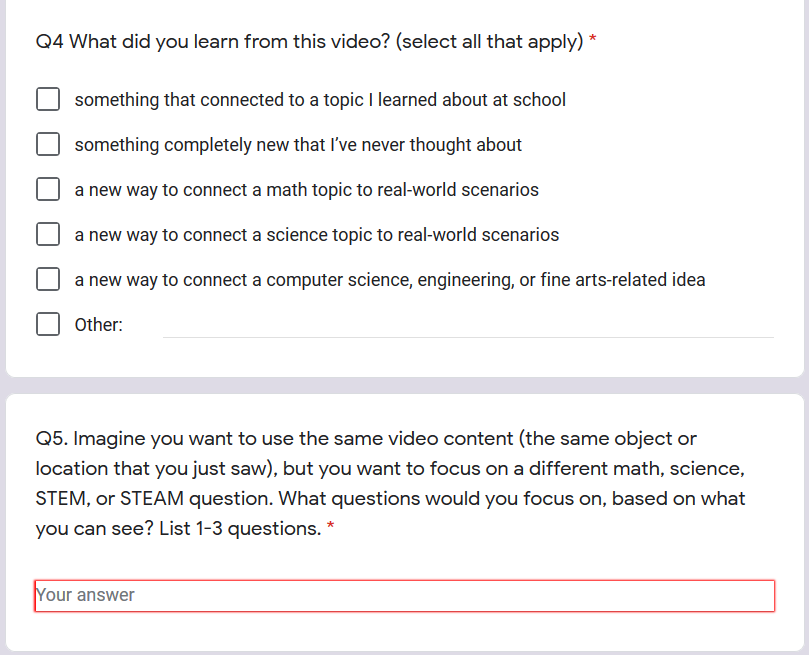

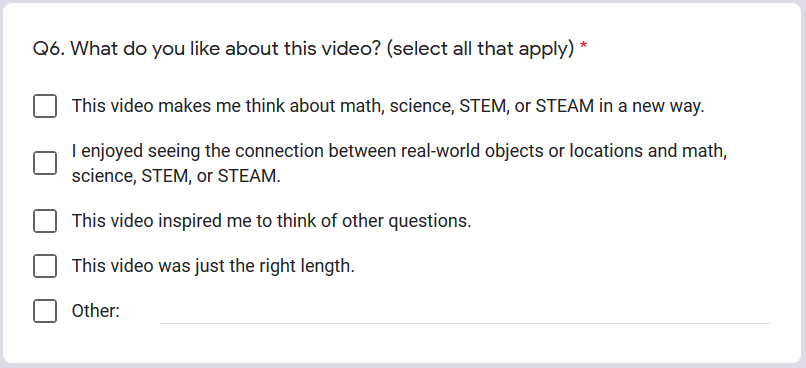


# Appendix B: Rubrics

## Appendix B1: WalkSTEM Project Rubric


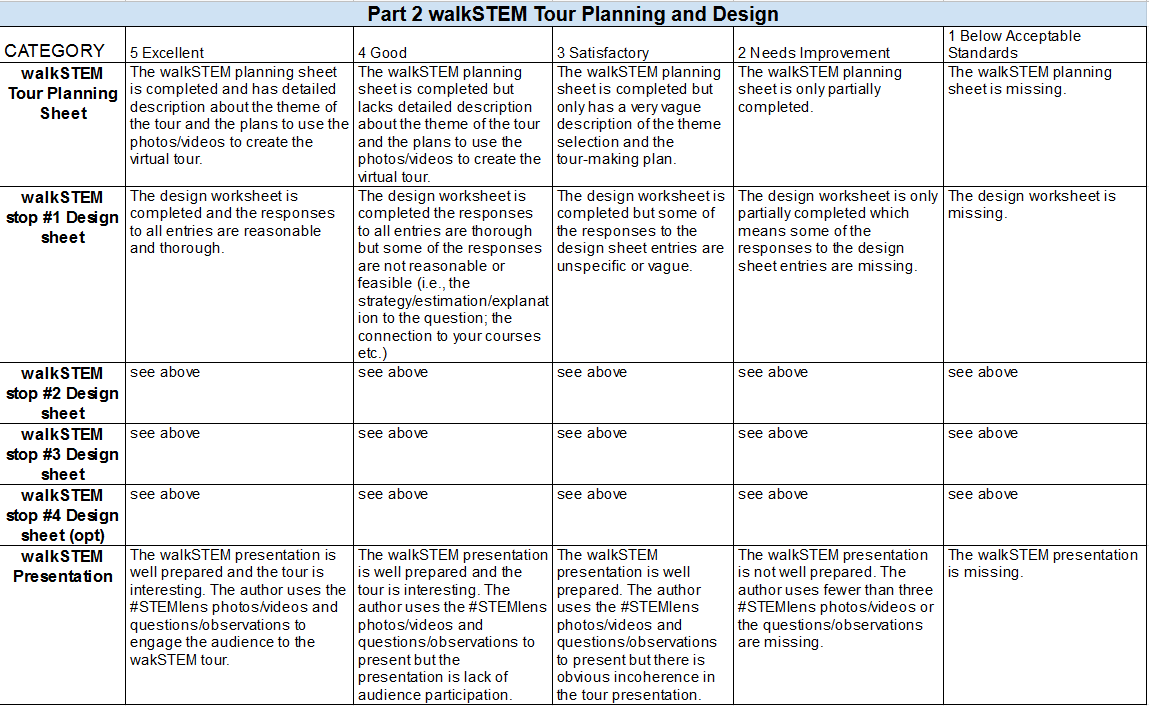

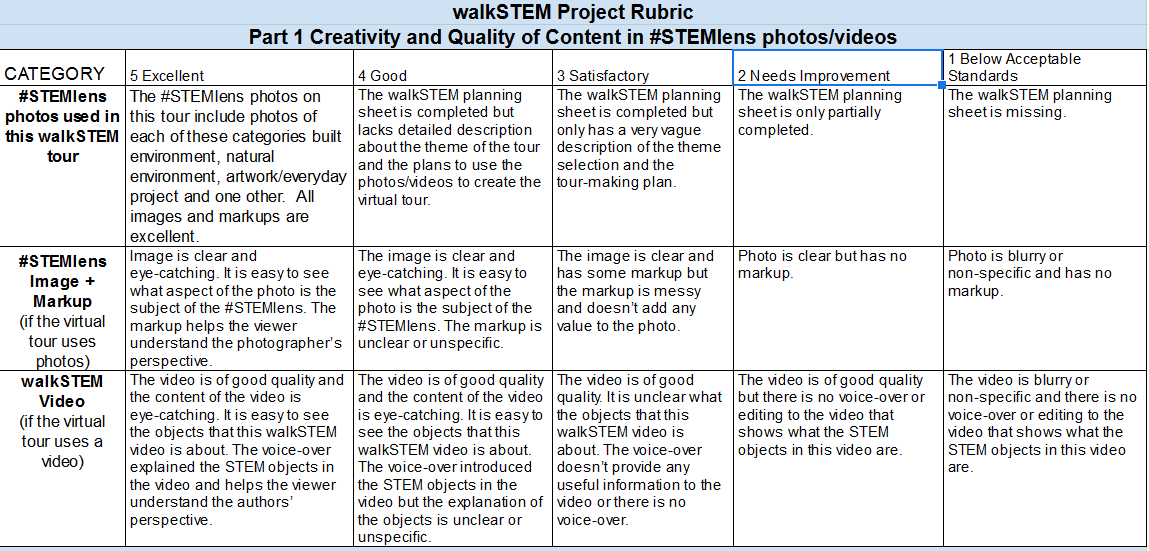


## Appendix B2: #STEMlens Rubric


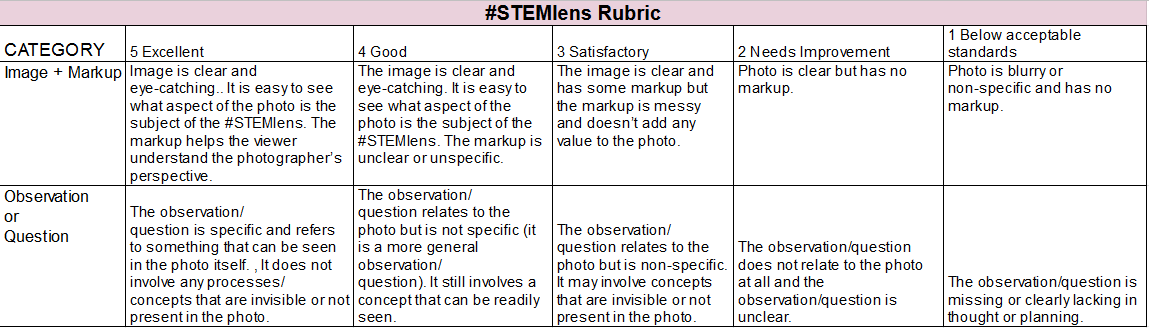


# Appendix C: Student Pre- and Post-Survey

1. **Pre- Only:**
2. What is your race/ethnicity?
3. What is your gender?
4. What languages are spoken in your home?
5. How old are you?
6. Which grade level are you in?
7. What school do you go to?
8. What math class or classes are you taking right now?
9. What grade do you typically make in your math classes?
10. What career are you interested in?
11. Do you plan to go to college? Where?
12. What do you plan to major in, when you go to college?
13. What’s your favorite subject in school?
14. What are some of your interests or hobbies?
15. Do you have any experience creating your own math problems?
16. Yes, I have created math problems before.
17. I have only created problems in other subjects, not math.
18. No, I don’t have experience in creating problems.
19. What experience do you have creating your own custom videos or with video editing?
20. **Pre- and Post**

|  | Strongly Disagree | Disagree | Neither Agree or Disagree | Agree | Strongly Agree |
| --- | --- | --- | --- | --- | --- |
| 1) Math is practical for me to know. |  |  |  |  |  |
| 2) Math helps me in my daily life outside of school. |  |  |  |  |  |
| 3) It is important for me to be a person who reasons mathematically. |  |  |  |  |  |
| 4) Thinking mathematically is an important part of who I am. |  |  |  |  |  |
| 5) I enjoy the subject of math. |  |  |  |  |  |
| 6) I like math. |  |  |  |  |  |
| 7) I enjoy doing math. |  |  |  |  |  |
| 8) Math is exciting to me. |  |  |  |  |  |

1. **Post- Only**
2. Do you like creating your own math problems in this program? Why or why not?
3. What suggestions do you have for teachers or students who are going to participate in future walkSTEM programs?
4. **Procedural Fluency, Conceptual Understanding, and Problem-Solving (pre- only)**
5. (Number, Item#M032094)

$$\frac{4}{100}+\frac{3}{1000}=$$

6. .043
7. .1043
8. .403
9. .43
10. (Number, Item#M032166)

Which of these is the BEST estimate of $\frac{7.21\times3.86}{10.09}?$

$\frac{7\times3}{10}$

$\frac{7\times4}{10}$

$\frac{7\times3}{11}$

$\frac{7\times4}{11}$

1. (Number, Item#M042002)

Place the four digits 3,5,7, and 9 into the boxes in the positions that would give the greatest result when the two numbers are multiplied.


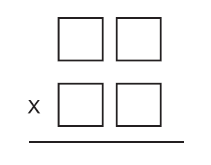


1. (Algebra, Item#M032205)

There were *m* boys and *n* girls in a parade. Each person carried 2 balloons. Which of these expressions represents the total number of balloons that were carried in the parade?

1. 2(*m* + *n*)
2. 2+(*m* + *n)*
3. 2*m* + *n*
4. *m* + 2*n*
5. (Algebra, Item#M032419)

Which of these could represent the expression 2*x* +3*x*

1. The length of this segment:
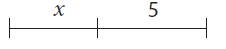

2. The length of this segment:
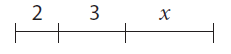

3.
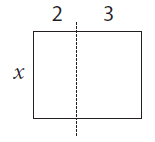
The length of this segment:


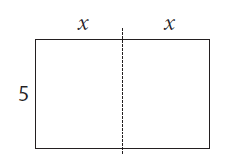


1. The length of this segment:
2. (Algebra, Item#M032424)

Jo has three metal blocks. The weight of each block is the same. When she weighed one block against 8 grams, this is what happened. When she weighed all three blocks against 20 grams, this is what happened.
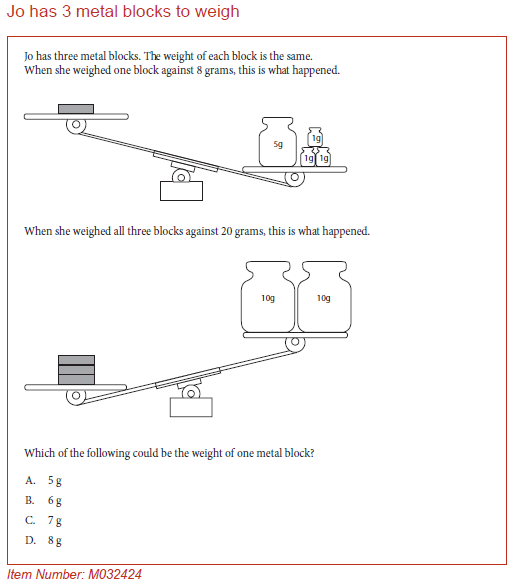

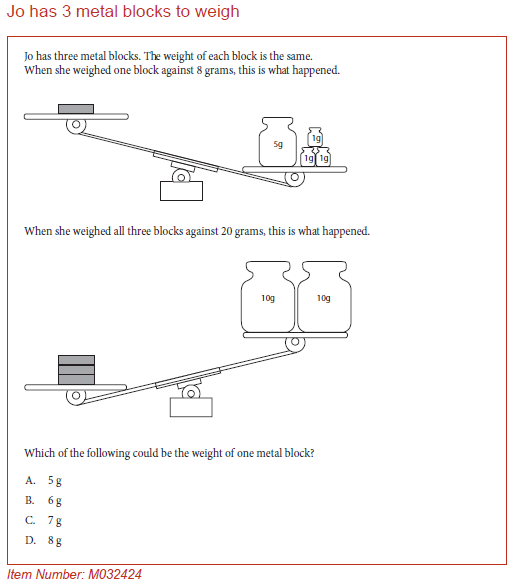


Which of the following could be the weight of one metal block?

1. 5g
2. 6g
3. 7g
4. 8g
5. (Geometry, Item#M042201)


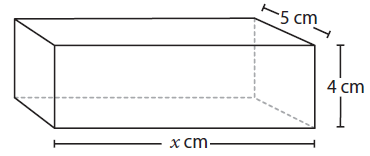


The volume of the rectangular box is 200 cm^3^. What is the value of *x*?

Answer: ___________

1. (Geometry, Item#M032402)


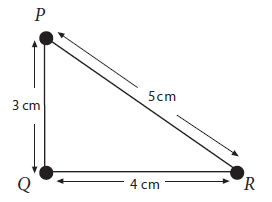


Which of these is the reason that triangle PQR is a right angle triangle?

1. 3^2^ + 4^2^ = 5^2^
2. 5 < 3+4
3. 3+4=12−5
4. 3 > 5−4
5. (Geometry, Item#M032100)

The figure above shows a shape made up of cubes that are all the same size. There is a hole all the way through the shape. How many cubes would be needed to fill the hole (Item#M032100)?


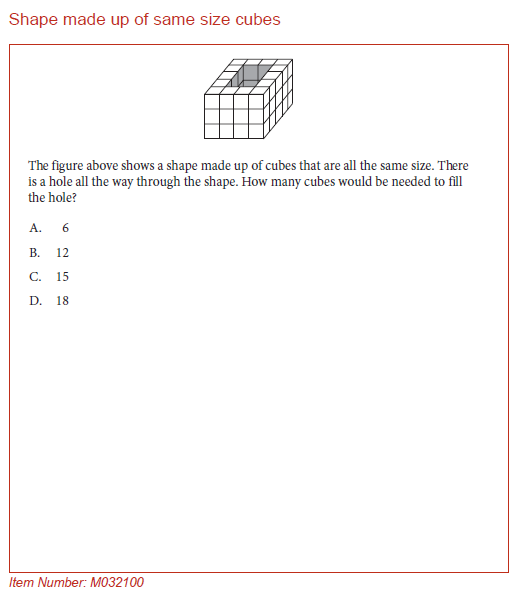


1. 6
2. 12
3. 15
4. 18
5. (Data)There are 25 girls in a class. The average height of the girls is 130 cm.
   1. (Item#M421Q01) Explain how the average height is calculated.
   2. (Item#M421Q02) Circle either “True” or “False” for each of the following statements.

| **Statement** | **True or False** |
| --- | --- |
| If there is a girl of height 132 cm in the class, there must be a girl of height 128 cm. | True/False |
| The majority of the girls must have height 130 cm. | True/False |
| If you rank all of the girls from the shortest to the tallest, then the middle one must have a height equal to 130 cm. | True/False |
| Half of the girls in the class must be below 130 cm, and half of the girls must be above 130 cm. | True/False |

10.3 (Item#M421Q03) An error was found in one student’s height. It should have been 120 cm instead of 145 cm. What is the correct average height of the girls in the class?

A. 126 cm

B. 127 cm

C. 128 cm

D. 129 cm

1. **Problem-Posing (pre- and post-)**

1) Describe the mathematical ideas you see in this picture. What questions might you pose based on this picture?


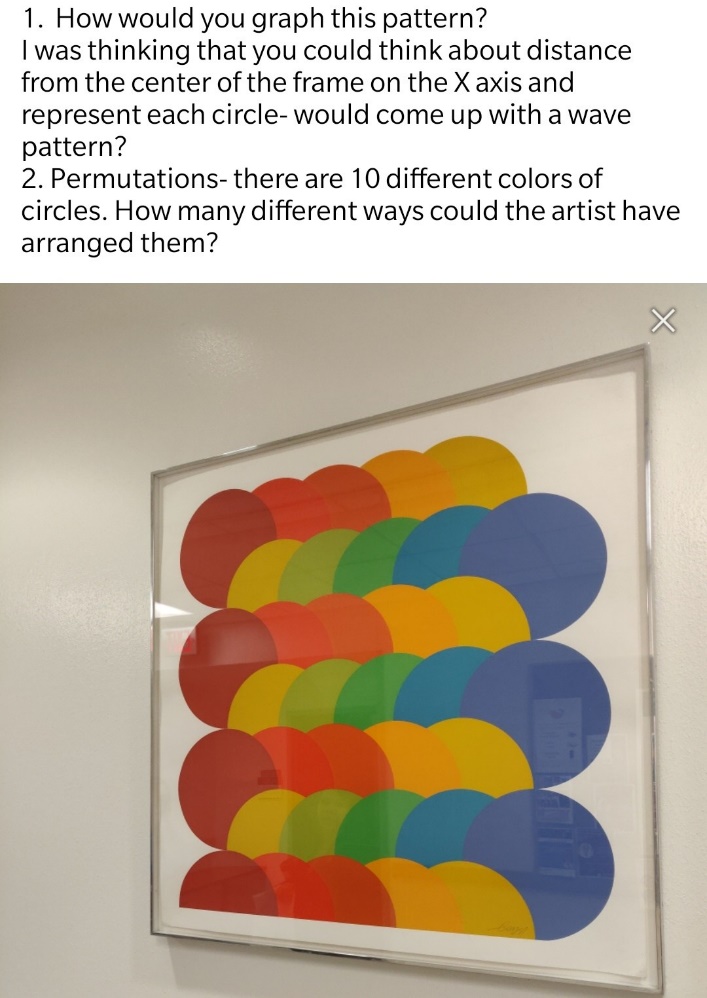


2) Describe how you see math in your home or neighborhood. Give at least 2 examples.

3) This is the plan of the apartment that George’s parents want to purchase from a real estate agency. Pose a mathematical problem based on this apartment floor plan or this apartment buying scenario.
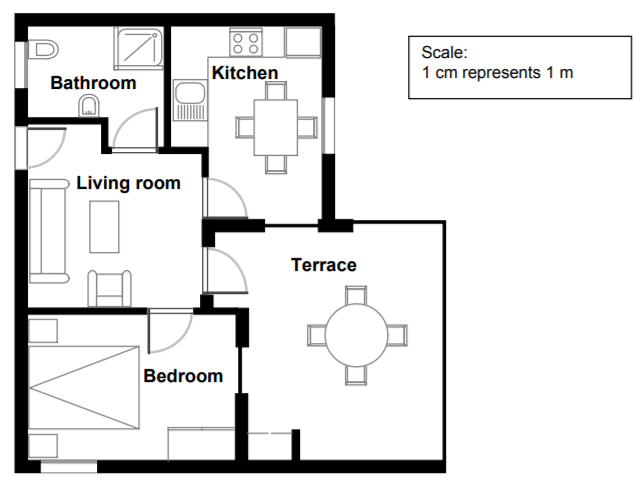


# Appendix D: Student Interview Protocol

I appreciate you letting me interview you for the research. I have some questions for you about the walkSTEM program that you attended. I will record this interview and the interview will only be used for this research.

- **Part 1: Background Information**
  - Which grade level are you in?
  - What’s your favorite subject in school?
  - What are some of your interests or hobbies?
  - Why did you choose to attend this walkSTEM program?
- **Part 2: Disposition Toward Problem Posing**
  - Do you have any experience of creating mathematical problems before attending this program?
  - How do you like the process that you create your own problems in this program?
  - What are some of the challenges that you encountered when creating your own problems?
  - Can you describe one specific example that you created a problem that you really liked creating in this program?
  - How do you think your experience of creating the math walk in this program will impact your mathematical learning in the future?
- **Part 3: About the online walkSTEM program**
  - How much do you like participating in the walkSTEM program?
  - How did you like the experience of creating your own virtual math walk?
  - What is the part that you like the most and the least in this program?
  - Compared to when you first began this program, has your interests or attitudes about mathematics changed? If so, can you explain how and why?
  - How do you think attending this walkSTEM program online instead of having in-person meetings has impact your experience of participating in these activities?
  - What suggestions do you have for this program if we are going to run this next year?
- **Part 4: Wrap Up**
  - Finally, is there anything else you want to share with me about your experience with this walkSTEM program?

Thank you so much for attending this interview! Your opinion is really important for our study.

# Appendix E: Potential Coding Foci

| Potential Coding Foci Related to Students’ Problem-Posing | |
| --- | --- |
| What topics do students focus on when they reflect on the video-watching task?  What topics do students focus on when discussing the #STEMlens photos?  What topics are their #STEMlens photos about?  What themes are mentioned when students are completing the walkSTEM planning sheet?  What topics do students focus on when creating the walkSTEM stops?  How do online tools (e.g., the gameboard, Zoom call, online searches) encourage or discourage student’s participation in the program? | |
| How do students interact with their peers? | |
| What STEM topics/concepts are covered in their discussion, #STEMlens photos, and walkSTEM projects? | |
| How do instructors scaffold students’ problem-posing?  What resources do students use to create their STEM walk? | |
